# Toxicities of Antibody–Drug Conjugates in Breast Cancer: From Mechanistic Insights to Clinical Management

**DOI:** 10.3390/pharmaceutics18070792

**Published:** 2026-06-28

**Authors:** Luisana Sisca, Mariam Grazia Polito, Arianna Travisani, Fernando Zannino, Michele Iuliani, Giuseppe Tonini, Francesco Pantano

**Affiliations:** 1 Department of Medical Oncology, Fondazione Policlinico Universitario Campus Bio-Medico, 00128 Rome, Italy; mariamgrazia.polito@unicampus.it (M.G.P.); arianna.travisani@unicampus.it (A.T.); fernando.zannino@unicampus.it (F.Z.); m.iuliani@policlinicocampus.it (M.I.); g.tonini@policlinicocampus.it (G.T.); f.pantano@policlinicocampus.it (F.P.); 2 Department of Translational Research, Institut Curie, 75005 Paris, France; 3 UOC Oncologia Territoriale-ASL Latina-CDS Aprilia, La Sapienza Università Di Roma Polo Pontino, 04100 Latina, Italy

**Keywords:** ADC, solid tumors, target selection, payload, resistance, tumor biology, breast cancer

## Abstract

**Background/Objectives:** Antibody–drug conjugates (ADCs) have transformed the therapeutic landscape of breast cancer, expanding treatment opportunities across multiple disease settings. However, their increasing clinical use has revealed a heterogeneous spectrum of toxicities that extends beyond conventional chemotherapy-related adverse events. Emerging evidence suggests that ADC-associated toxicities are driven by a complex interplay between ADC structural characteristics, including target antigen expression, payload properties, linker stability, drug-to-antibody ratio, and patient-related susceptibility factors. This review aims to provide a comprehensive overview of ADC-related toxicities in breast cancer, integrating mechanistic insights with clinical management strategies and risk-adapted approaches. **Methods:** A narrative review of the literature was conducted focusing on clinical trials, real-world studies, translational investigations, and mechanistic evidence related to ADC-associated toxicities in breast cancer. Particular attention was given to the relationship between ADC design, toxicity mechanisms, patient-specific risk factors, and clinical management. **Results:** ADC-related toxicities encompass a broad range of adverse events, including hematologic toxicity, interstitial lung disease, gastrointestinal complications, hepatotoxicity, peripheral neuropathy, stomatitis, ocular toxicity, dermatologic adverse events, and cardiovascular manifestations. Current evidence indicates that toxicity profiles differ substantially across ADCs and are influenced by multiple factors, including payload class, linker chemistry, target biology, intracellular trafficking, bystander effects, systemic payload exposure, and host-related characteristics. While several toxicities can be anticipated through careful monitoring and early intervention, clinically significant variability remains, and validated predictive biomarkers are largely lacking. Emerging real-world evidence further highlights the importance of individualized toxicity assessment and multidisciplinary management. **Conclusions:** ADC-related toxicity should be viewed as a multifactorial biological process resulting from the interaction between ADC design and host susceptibility rather than as a uniform class effect. A mechanistic understanding of toxicity pathways may improve risk stratification, toxicity monitoring, and personalized management strategies. Future research should focus on the development of predictive biomarkers, pharmacologic risk models, and next-generation ADC platforms with improved therapeutic indices. This review proposes an integrated framework linking ADC structural determinants, toxicity mechanisms, and clinical management to support safer and more individualized use of ADCs in breast cancer.

## 1. Introduction

Antibody–drug conjugates (ADCs) have emerged as a major therapeutic advancement in breast cancer, significantly improving the clinical outcomes across multiple disease settings. Agents such as trastuzumab emtansine (T-DM1), trastuzumab deruxtecan (T-DXd), and sacituzumab govitecan have demonstrated substantial efficacy, including in heavily pretreated populations, and are now integral components of modern treatment strategies [1].

However, the increasing use of ADCs has also highlighted a distinct and sometimes complex spectrum of treatment-related toxicities, some of which may be severe or potentially life-threatening. Among these, interstitial lung disease (ILD), hematologic toxicity, and gastrointestinal adverse events represent clinically relevant challenges that may limit treatment continuation and negatively impact patient outcomes.

While the biological mechanisms underlying ADC-related toxicities are partially understood, their occurrence remains only partially predictable, and optimal prevention and management strategies are still evolving. Notably, emerging evidence suggests that toxicity is not entirely random but may be influenced by a combination of patient-related characteristics, prior treatments, and drug-specific properties, including payload type and linker stability [2].

In this context, a more comprehensive understanding of the determinants of ADC-related toxicity is crucial to improve treatment safety and support individualized therapeutic approaches [3]. This review aims to provide a structured overview of ADC-related toxicities in breast cancer, with a specific focus on the clinical and biological risk factors, underlying mechanisms, and risk-adapted management strategies. Overall, current evidence suggests that ADC-related toxicity is multifactorial and influenced by a complex interaction between drug-specific and host-related factors, supporting the development of integrated risk stratification approaches.

## 2. Materials and Methods

This narrative review was conducted through a comprehensive literature search of the PubMed, Embase, and Scopus databases for articles published up to May 2026. The search strategy combined terms related to antibody–drug conjugates, breast cancer, toxicity, adverse events, interstitial lung disease, hematologic toxicity, gastrointestinal toxicity, risk factors, and toxicity management. Priority was given to pivotal phase II and phase III clinical trials, pooled safety analyses, regulatory documents, and international clinical practice guidelines when evaluating the incidence, mechanisms, and management of ADC-related toxicities. Real-world studies, pharmacovigilance analyses, and translational investigations were also considered to provide complementary information on toxicity patterns observed in routine clinical practice and to explore potential mechanistic determinants and risk factors. Additional references were identified through manual screening of the bibliographies of relevant publications. Given the narrative nature of this review, no formal systematic review protocol was applied. The objective was not to provide a quantitative synthesis of evidence, but rather to integrate mechanistic, clinical, and translational data into a comprehensive framework for understanding and managing ADC-related toxicities in breast cancer.

## 3. Overview of ADC Structure and Mechanisms of Toxicity

Antibody–drug conjugates (ADCs) are complex therapeutic agents composed of three essential components: a monoclonal antibody directed against a tumor-associated antigen, a cytotoxic payload, and a chemical linker connecting the two [4]. The therapeutic efficacy and safety profile of ADCs are determined by the interplay among these components, which collectively influence target specificity, intracellular drug delivery, and systemic exposure [5].

Following binding to the target antigen expressed on tumor cells, ADCs undergo receptor-mediated internalization and trafficking to lysosomes, where the linker is cleaved and the cytotoxic payload is released. Beyond target binding and payload release, several biological processes may influence ADC toxicity. Intracellular trafficking pathways determine the extent of payload processing and catabolite generation, while differences in payload permeability may affect diffusion into neighboring normal tissues. These factors contribute to variability in systemic exposure and off-target toxicity across different ADC platforms [6]. This process enables the selective delivery of highly potent cytotoxic agents to cancer cells, minimizing systemic toxicity compared to conventional chemotherapy. However, this selectivity is not absolute, and several mechanisms may contribute to off-target toxicity [7].

One key determinant of ADC behavior is the nature of the cytotoxic payload. Many ADCs used in breast cancer, such as trastuzumab deruxtecan (T-DXd) and sacituzumab govitecan, carry topoisomerase I inhibitors as payloads [8]. These agents are highly potent, and in some cases, membrane-permeable, allowing them to diffuse beyond the target cell and exert cytotoxic effects on neighboring cells, a phenomenon known as the “bystander effect”. While this property may enhance antitumor activity, it can also increase the risk of toxicity in normal tissues [9].

Another critical factor is the drug-to-antibody ratio (DAR), which represents the number of payload molecules attached to each antibody. T-DXd is characterized by a relatively high DAR compared to earlier ADCs, which may contribute to its increased efficacy, but also to a higher incidence of adverse events, including interstitial lung disease (ILD). The relationship between DAR and toxicity reflects the balance between therapeutic potency and systemic exposure [10].

Linker stability also plays a central role in determining ADC toxicity. Ideally, the linker should remain stable in circulation and release the payload only after internalization within tumor cells. However, premature cleavage of the linker may lead to systemic release of the cytotoxic agent, contributing to off-target toxicity. Differences in linker design across ADCs partly explain the variability in toxicity profiles observed among agents [11]. In addition to linker stability, conjugation technology may influence ADC pharmacokinetics and toxicity. Site-specific conjugation strategies can generate more homogeneous ADC populations and may reduce variability in payload distribution compared with conventional stochastic conjugation approaches [12].

Importantly, the expression of target antigens in normal tissues may also contribute to toxicity.

Although payload-mediated toxicity represents a major determinant of ADC safety, target biology should not be overlooked. HER2 and TROP2 are not exclusively expressed by malignant cells, and low-level expression in selected normal tissues may contribute to on-target, off-tumor toxicity. Consequently, the relative contribution of target-mediated and payload-mediated mechanisms may differ across ADC platforms and may partly explain the heterogeneity of organ-specific toxicity patterns observed in clinical practice. Further research is needed to better define the biological relevance of target expression in normal tissues and its contribution to ADC-associated adverse events. Consequently, ADC toxicity should be interpreted as the result of interactions among target biology, payload characteristics, linker chemistry, and host susceptibility rather than as a uniform class effect [13].

These pharmacological and biological differences translate into distinct toxicity profiles across ADCs. For example, T-DXd has been associated with ILD, a potentially severe and life-threatening adverse event, whereas sacituzumab govitecan is more commonly associated with hematologic toxicity and gastrointestinal adverse events, such as neutropenia and diarrhea. These differences highlight the importance of understanding ADC-specific properties when evaluating safety profiles [14].

**Figure 1:**
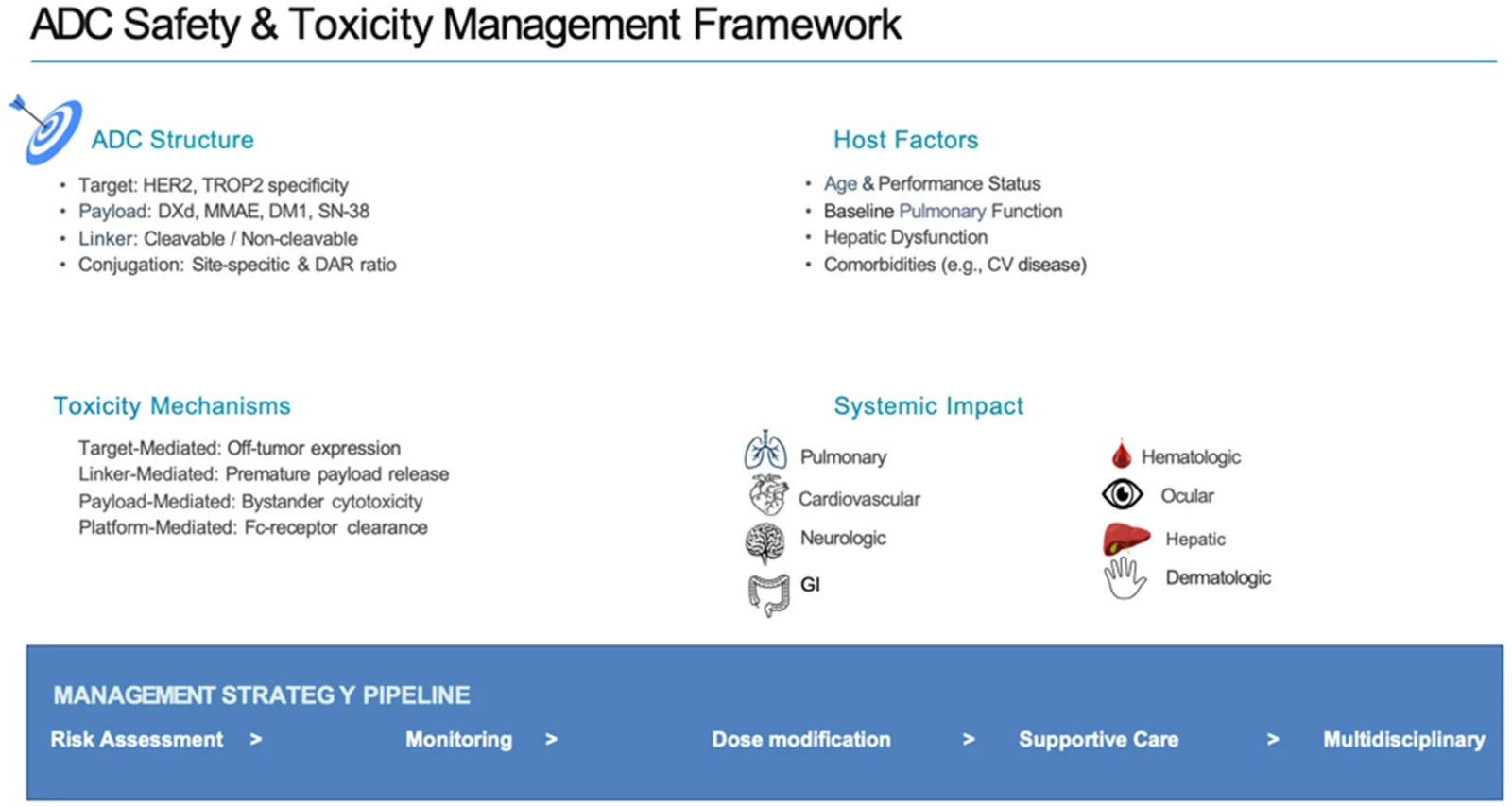
Determinants of ADC toxicity in breast cancer: from ADC design to clinical management. ADC-related toxicities arise from the interplay between structural features of antibody–drug conjugates (target antigen, payload characteristics, linker chemistry, drug-to-antibody ratio, and conjugation strategy) and host-related susceptibility factors. These determinants influence key biological processes, including target expression in normal tissues, Fc receptor-mediated uptake, intracellular trafficking, payload release, catabolite distribution, and bystander effects, ultimately leading to distinct toxicity mechanisms and organ-specific adverse events. Understanding these interactions may support risk stratification, toxicity monitoring, and personalized management strategies.

Overall, ADC-related toxicities arise from a complex interplay between drug design, target biology, and patient-specific factors (Figure 1). Although ADCs are often discussed as a therapeutic class, important differences exist among individual agents regarding target antigen, payload characteristics, and dominant toxicity profiles. The main mechanistic determinants and characteristic toxicity patterns of currently approved ADCs in breast cancer are summarized in Table 1. From a mechanistic perspective, ADC-related adverse events can be broadly categorized as target-mediated, payload-mediated, linker-mediated, or platform-mediated toxicities. Although these categories frequently overlap in clinical practice, this framework may facilitate interpretation of the heterogeneous toxicity profiles observed across different ADCs [15]. A deeper understanding of these mechanisms is essential to interpret variability in toxicity profiles and to support the development of predictive strategies aimed at optimizing treatment safety [16]. The relationship between ADC structural characteristics and their associated toxicity profiles is summarized in Table 2.

## 4. Major Toxicities of ADCs

ADC-related adverse events may arise through distinct but often overlapping biological mechanisms. For practical purposes, toxicities can be classified according to their predominant mechanistic drivers, including target-mediated, payload-mediated, linker-mediated, and platform-mediated effects, while host-related factors may further modulate toxicity risk and severity. The proposed framework is summarized in Table 3.

In this context, understanding why ADC-related toxicities differ across agents has become increasingly relevant for clinical practice. Emerging evidence indicates that toxicity profiles are shaped by a complex interaction between ADC structural characteristics, including target biology, payload properties, linker chemistry, drug-to-antibody ratio, conjugation strategy, and patient-specific susceptibility factors. Despite growing recognition of these mechanisms, important uncertainties remain regarding toxicity prediction, risk stratification, and optimal monitoring approaches.

This review provides a comprehensive and clinically oriented overview of ADC-related toxicities in breast cancer, integrating mechanistic determinants, host-related risk factors, toxicity patterns, and management strategies. Particular emphasis is placed on the biological mechanisms underlying toxicity heterogeneity across ADC platforms and on the development of a framework linking ADC design, toxicity mechanisms, and clinical decision-making.

### 4.1. Hematologic Toxicity

Hematologic toxicity, particularly neutropenia, represents a major adverse event associated with several antibody–drug conjugates (ADCs), with incidence and severity largely dependent on payload characteristics and systemic exposure [17].

Among the currently approved ADCs in breast cancer, sacituzumab govitecan is associated with the highest incidence of neutropenia. In the phase III ASCENT trial, grade ≥ 3 neutropenia was reported in approximately 51% of patients, with febrile neutropenia occurring in around 6% of cases [18]. These findings are consistent with the SN-38 payload, a topoisomerase I inhibitor known for its myelosuppressive effects. Similarly, in the TROPiCS-02 trial, sacituzumab govitecan demonstrated comparable rates of hematologic toxicity, confirming the reproducibility of this safety profile across different patient populations [19].

In contrast, trastuzumab deruxtecan (T-DXd) is associated with a lower incidence of severe neutropenia. In the DESTINY-Breast01 [20] and DESTINY-Breast03 [21] trials, grade ≥ 3 neutropenia was observed in approximately 15–20% of patients, while febrile neutropenia remained uncommon (<2%). Trastuzumab emtansine (T-DM1), on the other hand, is more frequently associated with thrombocytopenia rather than neutropenia, with grade ≥ 3 thrombocytopenia reported in up to 10–15% of patients in pivotal trials. These differences reflect the variability in payload type, drug-to-antibody ratio, and pharmacokinetic properties across ADCs, highlighting the importance of drug-specific risk assessment. From a clinical perspective, the use of granulocyte colony-stimulating factors (G-CSF) plays a key role in the prevention and management of neutropenia. According to current recommendations and drug-specific prescribing information, primary prophylaxis with G-CSF is not routinely required for all ADCs but should be considered in patients receiving sacituzumab govitecan who present with high-risk features, including prior extensive chemotherapy, baseline cytopenias, or advanced age. Secondary prophylaxis with G-CSF is recommended in patients who develop grade ≥ 3 neutropenia or febrile neutropenia during treatment. Both short-acting (e.g., filgrastim) and long-acting (e.g., pegfilgrastim) G-CSF formulations may be used. Short-acting G-CSF is typically administered daily until neutrophil recovery, whereas long-acting formulations are administered once per cycle and may improve patient convenience and adherence. The choice between short- and long-acting formulations should be individualized based on patient characteristics, treatment schedule, and institutional practice. Dose modifications remain an essential component of hematologic toxicity management. Treatment interruption and dose reduction, as recommended in the prescribing information for each ADC, are critical to ensure patient safety while maintaining therapeutic efficacy [22,23].

Overall, hematologic toxicity associated with ADCs may be partially anticipated on the basis of drug-specific and patient-related risk factors and may be mitigated through appropriate supportive care and monitoring strategies. In this context, a proactive approach integrating patient-related risk factors, drug-specific toxicity profiles, and early use of G-CSF may significantly improve treatment tolerability [24].

Importantly, according to current international guidelines (e.g., ESMO and ASCO), the use of G-CSF should be guided by the overall risk of febrile neutropenia, integrating both treatment-related and patient-specific factors. Consistently, real-world evidence from retrospective cohorts, including studies by Tam et al. [25] and the multicenter SACISUR cohort [26] has shown rates of neutropenia comparable to those observed in clinical trials, further confirming the overall consistency of hematologic toxicity patterns observed in clinical trials and routine clinical practice. Despite the overall consistency between clinical trials and real-world evidence, some uncertainties remain. In particular, the impact of cumulative exposure, prior lines of therapy, and interindividual variability in drug metabolism on hematologic toxicity risk is not fully elucidated. Furthermore, real-world populations may include more fragile patients, potentially leading to a higher incidence of severe events than that observed in controlled trial settings.

### 4.2. Interstitial Lung Disease (ILD)

The pathogenesis of ADC-related interstitial lung disease (ILD), particularly in the context of trastuzumab deruxtecan (T-DXd), is complex and not yet fully elucidated. Current evidence supports a multifactorial model involving direct cytotoxic injury, immune-mediated mechanisms, and patient-specific susceptibility factors [27].

A central mechanism is thought to be off-target toxicity related to the premature release of the cytotoxic payload. T-DXd delivers a membrane-permeable topoisomerase I inhibitor, which can diffuse beyond tumor cells and reach adjacent normal tissues, including the pulmonary interstitium. While this bystander effect may enhance antitumor efficacy, it may also result in the unintended exposure of alveolar epithelial cells to cytotoxic agents, leading to cellular injury and apoptosis.

In addition to passive diffusion, uptake of ADCs by non-malignant cells has been proposed as a relevant mechanism. Preclinical studies suggest that alveolar macrophages may internalize ADCs through Fc receptor-mediated pathways, resulting in intracellular release of the payload and local inflammatory activation. This process may contribute to the initiation and amplification of lung injury, potentially progressing from subclinical inflammation to clinically significant pneumonitis.

The physicochemical properties of the ADC also play a critical role. The relatively high drug-to-antibody ratio (DAR) of T-DXd increases the amount of cytotoxic payload delivered per molecule, potentially enhancing both efficacy and systemic exposure. Moreover, linker stability represents a key determinant of toxicity, as premature cleavage in circulation may lead to systemic release of the payload, increasing the risk of off-target damage, including in lung tissue [28].

An immune-mediated component is also likely involved. ADC-induced tissue injury may trigger local cytokine release and immune cell recruitment, resulting in an inflammatory cascade that amplifies lung damage. The clinical effectiveness of corticosteroids in managing ADC-related ILD supports the hypothesis of an inflammatory or immune-driven component in at least a subset of cases [29].

From a clinical perspective, several patient-related factors have been associated with an increased risk of ILD, including older age, pre-existing lung disease, and prior exposure to lung-toxic treatments such as thoracic radiotherapy. Geographic and ethnic differences have also been observed, with a higher incidence reported in Asian populations. Treatment-related factors, including longer duration of therapy and cumulative exposure, may further influence ILD risk, suggesting a possible dose–toxicity relationship [30].

Clinically, the reported incidence of ILD with trastuzumab deruxtecan ranges from approximately 10% to 15% across clinical trials, with grade ≥ 3 events occurring in 1–3% of patients and a small but clinically significant risk of fatal outcomes (approximately 1–2%). These findings underscore the importance of early detection and prompt management. Emerging biomarkers, such as KL-6 and surfactant proteins (SP-A, SP-D), are currently being investigated, although their clinical role remains to be established [31].

From a management perspective, a grade-based approach is recommended. In asymptomatic (grade 1) cases, treatment interruption and close monitoring are advised. In contrast, symptomatic ILD (grade ≥ 2) requires permanent discontinuation of the ADC and prompt initiation of systemic corticosteroids, with early intervention being associated with improved clinical outcomes [32].

Importantly, real-world data have largely confirmed the incidence of ILD observed in clinical trials. Large observational cohorts, such as the EN-SEMBLE study by Nozawa et al. [33], as well as post-marketing surveillance studies from Japan [34] have reported comparable incidence rates in routine clinical practice. Additional real-world analyses, including the study by Udovica et al., suggest that while overall ILD incidence is similar to that reported in clinical trials, the proportion of high-grade events may be higher in real-world settings, highlighting the importance of early detection and proactive management [35]. Real-world evidence on ADC-related toxicities across different clinical settings is summarized in Table 4.

In this context, recent multicenter analyses presented at international congresses, including ESMO Breast Cancer 2026 (Gullotta et al.) [40], have provided additional insights into cumulative ILD incidence and risk stratification in routine clinical practice, reinforcing the role of both patient- and treatment-related determinants. Despite the growing body of evidence, several critical gaps remain. The relative contribution of drug-related versus patient-related risk factors is not fully defined, and the absence of validated predictive biomarkers limits the ability to identify high-risk patients before treatment initiation. Moreover, real-world data suggest that the incidence and severity of ILD may be higher than reported in clinical trials, possibly reflecting broader patient selection and less stringent monitoring. These considerations highlight the need for standardized surveillance strategies and prospective studies aimed at improving risk stratification.

### 4.3. Gastrointestinal Toxicity

Gastrointestinal adverse events, including nausea, vomiting, and diarrhea, are commonly observed with antibody–drug conjugates (ADCs), particularly those carrying topoisomerase I inhibitor payloads. These toxicities may significantly impact quality of life, treatment adherence, and dose intensity, and are largely related to systemic exposure to the cytotoxic payload and off-target effects on rapidly proliferating gastrointestinal epithelial cells [41].

Among the ADCs used in breast cancer, sacituzumab govitecan is most strongly associated with gastrointestinal toxicity. In the phase III ASCENT trial [42], diarrhea of any grade was reported in approximately 60–65% of patients, with grade ≥ 3 events occurring in around 10–12%. Nausea was reported in more than 50% of patients, while vomiting occurred in approximately 30% of cases. These findings are consistent with the SN-38 payload, a topoisomerase I inhibitor known to induce gastrointestinal mucosal damage through the inhibition of epithelial cell turnover [43].

Similarly, trastuzumab deruxtecan (T-DXd), which also carries a topoisomerase I inhibitor payload, is associated with gastrointestinal adverse events, although generally at lower incidence and severity compared to sacituzumab govitecan. In DESTINY-Breast trials, nausea has been reported in up to 70–75% of patients, mostly grade 1–2, while grade ≥ 3 nausea and vomiting remain uncommon (<5%). Diarrhea is less frequent but still clinically relevant. In contrast, trastuzumab emtansine (T-DM1), which carries a microtubule inhibitor payload (DM1), is associated with a lower incidence of gastrointestinal toxicity, highlighting the role of payload type in determining toxicity profiles [44].

Several patient-related factors may influence the risk and severity of gastrointestinal toxicity. These include prior exposure to chemotherapy, baseline gastrointestinal disorders, performance status, and individual variability in drug metabolism. In the case of sacituzumab govitecan, polymorphisms in UGT1A1 (particularly UGT1A1 *28/*28 genotype) have been associated with an increased risk of neutropenia and diarrhea, reflecting impaired metabolism of SN-38. This represents one of the few clinically relevant pharmacogenomic markers in the context of ADC therapy. Although routine testing is not universally recommended, it may be considered in selected patients to better inform risk assessment and supportive care strategies [45].

From a supportive care perspective, both nutritional and pharmacologic strategies play an important role. Although high-level evidence is limited, adequate hydration, small frequent meals, and avoidance of irritant foods may help reduce symptom burden. In patients with diarrhea, low-residue diets and electrolyte supplementation may be beneficial, although formal dietary recommendations are largely extrapolated from conventional chemotherapy management.

In parallel, pharmacologic supportive care represents the cornerstone of management. Early initiation of antidiarrheal agents such as loperamide is recommended at the onset of symptoms and remains the standard first-line approach, particularly in patients receiving sacituzumab govitecan, in whom prompt intervention is essential due to the higher incidence and potential severity of diarrhea. In selected cases of persistent or refractory diarrhea, additional supportive strategies have been explored. Rifaximin, a non-absorbable antibiotic, has been investigated in chemotherapy-induced diarrhea; however, ADC-specific evidence remains extremely limited, and its routine use cannot currently be recommended.

Similarly, modulation of the gut microbiota through probiotics has been proposed as a potential supportive approach. Nevertheless, available evidence is heterogeneous, largely derived from non-ADC settings, and insufficient to support formal recommendations in patients receiving ADC therapy. Further prospective studies are needed to clarify the potential role of these interventions [46].

Overall, the management of gastrointestinal toxicity requires a proactive and symptom-driven approach. Antiemetic prophylaxis is recommended, particularly for ADCs associated with moderate to high emetogenic risk such as T-DXd. Standard regimens, including serotonin (5-HT3) receptor antagonists, dexamethasone, and in selected cases, NK1 receptor antagonists, may be used according to established antiemetic guidelines. More specifically, antiemetic prophylaxis should be administered in accordance with international guidelines (ESMO/MASCC/ASCO), taking into account both the emetogenic potential of the ADC and individual patient risk factors [47].

For diarrhea, early initiation of antidiarrheal agents such as loperamide is essential, while refractory cases may require treatment interruption and further evaluation. Dose modifications remain a key component of toxicity management; according to prescribing information, treatment should be withheld in patients experiencing grade ≥ 3 gastrointestinal toxicity until resolution to grade ≤ 1. Dose reduction should be considered upon re-treatment, particularly in cases of recurrent or persistent toxicity, whereas permanent discontinuation may be necessary in rare cases of severe or unmanageable adverse events [48].

Overall, gastrointestinal toxicity associated with ADCs is strongly influenced by payload characteristics and may be reduced through early symptom recognition, supportive care, and timely dose modification. A personalized approach integrating patient-related risk factors, early symptom recognition, and supportive strategies, including nutritional interventions and microbiota modulation, is essential to optimize treatment tolerability and maintain therapeutic efficacy. In this context, real-world experience suggests that gastrointestinal toxicities are common but generally manageable, although observational data indicate a potentially greater impact on patient-reported quality of life compared to clinical trial reporting. However, despite the availability of effective supportive strategies, gastrointestinal toxicity remains a relevant cause of dose reduction and treatment discontinuation in clinical practice. In addition, the role of pharmacogenomic profiling, such as UGT1A1 testing, remains not fully standardized, and its integration into routine clinical decision-making is still debated. Further studies are warranted to better define personalized approaches to prevention and management [49].

### 4.4. Ocular Toxicity

Ocular adverse events have been increasingly recognized as a relevant toxicity associated with specific antibody–drug conjugates (ADCs), although their incidence and severity vary widely depending on drug- and payload-related characteristics. These toxicities are generally considered drug-specific and are likely related to off-target uptake, local tissue susceptibility, and the exposure of corneal epithelial cells to cytotoxic payloads [50].

Among the ADCs, ocular toxicity has been most extensively described with agents carrying microtubule inhibitor payloads. However, it remains relatively uncommon in breast cancer ADCs such as trastuzumab deruxtecan (T-DXd), sacituzumab govitecan, and trastuzumab emtansine (T-DM1) [51]. In clinical trials evaluating T-DXd, ocular adverse events, including dry eye and blurred vision, have been reported in approximately 10–20% of patients, with most events being grades 1–2 and rarely leading to treatment discontinuation. Similarly, sacituzumab govitecan has been associated with low rates of ocular toxicity, generally below 10%, and typically mild in severity [52].

In contrast, higher rates of ocular toxicity have been reported with ADCs outside the breast cancer setting, such as mirvetuximab soravtansine, suggesting that both payload characteristics and target expression may significantly influence ocular risk. These observations support the hypothesis that corneal epithelial cells may be particularly susceptible to certain cytotoxic payloads, especially in the presence of membrane permeable compounds [53].

The pathophysiology of ADC related ocular toxicity is not fully understood. Proposed mechanisms include direct exposure of the corneal epithelium to circulating cytotoxic payload, uptake of ADCs by rapidly dividing epithelial cells, and local inflammatory responses. Unlike ILD, ocular toxicity does not appear to involve a significant immune mediated component but rather reflects the local susceptibility of ocular surface tissues [54].

Data on risk factors remain limited. However, baseline ocular conditions, including dry eye syndrome or prior ocular surface disease, may increase susceptibility. Advanced age and cumulative exposure to ADC therapy may also contribute, although available evidence is inconsistent. In addition, interindividual variability in drug distribution and metabolism may further influence toxicity risk [55].

Real-world data are currently limited but appear to confirm the generally low incidence and mild severity of ocular toxicity in breast cancer populations treated with currently approved ADCs. Nevertheless, underreporting of low-grade symptoms, such as dry eye or visual discomfort, cannot be excluded.

From a clinical perspective, management is largely supportive and based on early recognition and symptom control. Preventive strategies include patient education, and in selected cases, baseline ophthalmologic assessment. The use of lubricating eye drops (artificial tears) is commonly recommended to reduce symptoms and maintain corneal integrity. In patients with more persistent symptoms, topical anti-inflammatory agents or corticosteroid eye drops may be considered, although evidence remains limited and should be individualized [56].

Dose modifications are rarely required but may be considered in cases of persistent or worsening symptoms. Temporary treatment interruption is generally sufficient, and most ocular adverse events resolve with supportive care.

Overall, ocular toxicity associated with ADCs in breast cancer is typically mild and rarely dose-limiting, although early recognition and supportive interventions remain important to minimize symptom burden [57]. It should be acknowledged that much of the current mechanistic understanding of ADC-associated ocular toxicity derives from agents developed outside the breast cancer setting, particularly mirvetuximab soravtansine and other ADCs carrying microtubule inhibitor payloads. Therefore, extrapolation of these findings to breast cancer ADCs should be performed with caution. Available evidence from trastuzumab deruxtecan, trastuzumab emtansine, and sacituzumab govitecan suggests a substantially lower incidence and severity of ocular adverse events, highlighting the potential influence of ADC-specific structural and pharmacologic characteristics. Nevertheless, increased awareness of these events and early supportive interventions are essential to prevent symptom progression and maintain treatment adherence. Nevertheless, the limited availability of real-world data and the potential underreporting of low-grade symptoms represent important limitations. Further prospective studies are needed to better characterize the true incidence, underlying mechanisms, and optimal preventive strategies for ADC-related ocular toxicity.

### 4.5. Stomatitis

Stomatitis has emerged as a relevant toxicity associated with newer antibody–drug conjugates (ADCs), particularly agents such as datopotamab deruxtecan. Although these events are typically low-grade, they may nonetheless have a clinically meaningful impact on patient quality of life, and in some cases, lead to treatment interruptions or dose modifications [58].

Emerging evidence suggests that stomatitis may reflect drug-specific properties, potentially related to the cytotoxic payload and its effects on rapidly proliferating mucosal epithelial cells. In addition, local tissue susceptibility may further contribute to the development and severity of these events.

Overall, while generally manageable, stomatitis represents an additional component of the evolving toxicity profile of next-generation ADCs and warrants clinical awareness, particularly as these agents are increasingly used in broader treatment settings [59].

### 4.6. Cardiovascular Toxicity

Cardiovascular toxicity has been historically associated with HER2-targeted therapies; however, its role in the context of antibody–drug conjugates (ADCs) remains less well-defined and is likely underrecognized. While clinical trials have generally reported a low incidence of major cardiovascular events with ADCs such as trastuzumab emtansine (T-DM1) and trastuzumab deruxtecan (T-DXd), the increasing use of these agents in broader and more heterogeneous patient populations has raised concerns regarding their cardiovascular safety profile [60].

The mechanisms underlying ADC-related cardiovascular toxicity are likely multifactorial. HER2 signaling plays a critical role in cardiomyocyte survival and stress adaptation, and its inhibition may contribute to left ventricular dysfunction, as observed with conventional HER2-targeted therapies. In addition, ADC-specific properties may further modulate cardiovascular risk. These include systemic exposure to cytotoxic payloads, which may induce endothelial injury, oxidative stress, and mitochondrial dysfunction, as well as potential pro-thrombotic effects related to circulating drug metabolites. Off target uptake of ADCs by non-malignant tissues and variability in linker stability may also contribute to unintended cardiovascular exposure [61].

Patient-related factors are also likely to play an important role in determining susceptibility to cardiovascular adverse events. These include age, baseline cardiovascular comorbidities, and prior exposure to cardiotoxic treatments such as anthracyclines or radiotherapy [62].

In this context, emerging real-world pharmacovigilance data have provided additional insights into the cardiovascular safety profile of ADCs. Analyses based on large databases such as the FDA Adverse Event Reporting System (FAERS), including the study by Hu et al., have identified potential safety signals for specific cardiovascular events, including major adverse cardiovascular events (MACE) and stroke, particularly in younger patients. These findings suggest that ADC-related cardiovascular toxicity may not be entirely negligible and may involve complex interactions between drug-related and host-related factors [63]. These findings should be interpreted with caution and considered hypothesis-generating rather than evidence of causal associations, given the intrinsic limitations of spontaneous pharmacovigilance databases.

From a clinical perspective, the spectrum of cardiovascular adverse events associated with ADCs appears heterogeneous, ranging from asymptomatic declines in left ventricular ejection fraction to more acute events such as ischemic or thromboembolic complications. However, current evidence remains limited, and the true incidence of these events in routine clinical practice is not fully established.

In the absence of ADC-specific guidelines, management strategies are largely extrapolated from established approaches used for HER2-targeted therapies. Baseline cardiovascular assessment, including the evaluation of cardiac function and risk factors, may be considered prior to treatment initiation. During therapy, periodic monitoring of cardiac function may be warranted, particularly in patients at higher risk [64].

In cases of suspected cardiotoxicity, treatment interruption and cardiology consultation are recommended. Standard cardioprotective therapies, including beta-blockers and angiotensin-converting enzyme inhibitors, may be initiated when appropriate. Permanent discontinuation of ADC therapy may be required in patients with severe, symptomatic, or persistent cardiovascular toxicity [65].

Overall, although cardiovascular toxicity does not currently represent a predominant limitation of ADC therapy, increasing real-world evidence suggests that it should be considered in the overall safety assessment, particularly as ADCs are used in earlier treatment settings and in more heterogeneous patient populations. Further prospective and real-world studies are needed to better define its incidence, underlying mechanisms, and optimal management. Notably, cardiovascular toxicity may be underrecognized and potentially underreported in clinical trials, where patients with significant baseline comorbidities are often excluded. As a result, the true incidence of cardiovascular events in real-world populations may be higher than currently appreciated. This discrepancy underscores the need for dedicated prospective studies and more systematic cardiovascular monitoring strategies in patients receiving ADCs.

### 4.7. Alopecia and Supportive Strategies

Alopecia is not a predominant toxicity across all antibody–drug conjugates (ADCs) but may be clinically relevant with specific agents, particularly sacituzumab govitecan. In clinical trials, alopecia has been reported in approximately 40–50% of patients treated with this agent, likely reflecting the effects of its SN-38 payload [2] on rapidly proliferating hair follicle cells [18]. In contrast, trastuzumab deruxtecan appears to be associated with a lower incidence of alopecia, generally below 20% [20], suggesting that differences in payload characteristics, systemic exposure, and pharmacokinetics may influence this toxicity. Although rarely life-threatening, alopecia can have a substantial impact on patient quality of life, affecting body image, psychological well-being, and treatment perception, and may contribute to treatment-related distress. Scalp cooling is an established strategy for the prevention of chemotherapy-induced alopecia, acting through vasoconstriction and reduced drug delivery to hair follicles, with studies reporting a reduction in alopecia risk of approximately 40–50% [66]. However, its role in patients receiving ADCs remains uncertain. Unlike conventional chemotherapy, ADC-related alopecia may result from off-target exposure to cytotoxic payloads released systemically or via the bystander effect, mechanisms that may not be fully mitigated by reduced scalp perfusion. To date, no prospective data have specifically evaluated scalp cooling in ADC-treated populations, and its clinical benefit in this setting remains to be established.

### 4.8. Hepatotoxicity

Hepatotoxicity represents a clinically relevant but often underrecognized adverse event associated with antibody–drug conjugates (ADCs), particularly trastuzumab emtansine (T-DM1). The most commonly reported manifestations include elevations of alanine aminotransferase (ALT), aspartate aminotransferase (AST), and bilirubin levels, although severe hepatic dysfunction is uncommon. In pivotal clinical trials, hepatic laboratory abnormalities were generally manageable; however, post-marketing experience has highlighted rare but clinically significant complications, including nodular regenerative hyperplasia (NRH) and non-cirrhotic portal hypertension. The mechanisms underlying ADC-related hepatotoxicity are incompletely understood and are likely multifactorial. Potential contributors include off-target uptake by hepatic cells, intracellular accumulation of cytotoxic catabolites, and payload-mediated cellular injury. The liver also plays a central role in ADC metabolism and clearance, potentially increasing susceptibility to toxic exposure. In addition, prolonged treatment duration and cumulative exposure may further contribute to hepatic injury [67]. From a clinical perspective, most cases are asymptomatic and identified through routine laboratory monitoring. Nevertheless, persistent elevations of liver enzymes or bilirubin may require treatment interruption, dose reduction, or permanent discontinuation according to prescribing information. Particular attention should be paid to patients with pre-existing liver disease or concomitant hepatotoxic medications. Baseline assessment of liver function and periodic monitoring throughout treatment are therefore recommended. Early identification of hepatic abnormalities may facilitate timely intervention and minimize treatment-related complications.

### 4.9. Peripheral Neuropathy

Peripheral neuropathy is a recognized toxicity associated with ADCs carrying microtubule inhibitor payloads and represents a classic example of payload-mediated toxicity. The pathogenesis resembles that observed with conventional microtubule-targeting agents and is thought to result from the disruption of axonal transport and neuronal microtubule function. Although less common than hematologic or gastrointestinal adverse events, peripheral neuropathy may substantially affect quality of life, daily functioning, and long-term treatment adherence. Clinical manifestations typically include paresthesia, numbness, tingling, sensory disturbances, and less frequently, motor impairment. Symptoms often develop gradually and may worsen with cumulative exposure. The incidence and severity of neuropathy vary according to ADC structure, payload characteristics, treatment duration, and individual patient susceptibility. Patients with pre-existing neuropathy, diabetes mellitus, or prior exposure to neurotoxic therapies may be at increased risk. Management relies primarily on early recognition, routine neurologic assessment, and timely dose modification. Treatment interruption and dose reduction remain the mainstay of management, while supportive interventions may be considered for persistent symptoms. Although many cases improve following treatment adjustment, complete recovery may be delayed, emphasizing the importance of early intervention [68].

### 4.10. Infusion-Related Reactions

Infusion-related reactions (IRRs) have been reported with several ADCs, although their incidence remains relatively low compared with other treatment-related toxicities. Clinical manifestations may include fever, chills, flushing, rash, dyspnea, hypotension, and other hypersensitivity-like symptoms occurring during or shortly after drug administration. The underlying mechanisms are not fully elucidated but may involve cytokine release, immune activation, or reactions to ADC components. Most events are grades 1–2 and occur during the first infusion. Severe reactions are uncommon but require prompt recognition and management. Clinical management includes temporary interruption of the infusion, supportive measures, and symptomatic treatment. In selected cases, slower infusion rates or premedication may be considered. Permanent discontinuation is rarely necessary, and most patients can successfully continue treatment following appropriate management [69].

### 4.11. Fatigue

Fatigue is among the most frequently reported adverse events across multiple ADCs, although it is often underrepresented in mechanistic discussions due to its multifactorial nature. The etiology is likely complex and may involve treatment-related inflammation, anemia, nutritional status, sleep disturbances, psychological stress, and cumulative treatment burden. Although fatigue is generally low grade, it may significantly impair quality of life, physical functioning, and treatment adherence. Importantly, patient-reported outcomes often suggest a greater impact than that captured through conventional adverse-event reporting systems. Management is primarily supportive and should include the assessment of potentially reversible causes, optimization of comorbid conditions, nutritional support, physical activity when feasible, and patient education. A multidisciplinary approach may be particularly beneficial in patients experiencing persistent or severe symptoms [70].

### 4.12. Dermatologic Toxicities

Dermatologic adverse events have been increasingly recognized with ADC therapy and may include rash, pruritus, xerosis, erythema, pigmentary alterations, and nail changes. Although these toxicities are generally mild to moderate in severity, they may substantially affect patient comfort and quality of life. The biological mechanisms remain incompletely understood but likely involve off-target exposure of rapidly proliferating epithelial tissues to cytotoxic payloads. In addition, local inflammatory responses and individual susceptibility factors may contribute to clinical manifestations. Most dermatologic toxicities can be effectively managed through patient education, skin hydration, avoidance of irritants, and topical therapies when appropriate. Early dermatologic consultation may be beneficial in persistent or severe cases. Treatment interruption or dose modification is rarely required but may be considered when symptoms significantly affect quality of life or fail to respond to supportive measures. Overall, increased awareness and proactive management of dermatologic adverse events may improve treatment tolerability and support long-term adherence to ADC therapy [71].

## 5. Determinants of Toxicity Risk and Clinical Implications

Patient-specific factors, including organ function, comorbidities, and prior treatments, may contribute substantially to variability in toxicity risk [72].

Overall, risk factors for ADC-related toxicity can be broadly categorized into three groups: (i) patient-related factors (e.g., age, comorbidities, organ function), (ii) treatment-related factors (e.g., prior therapies, cumulative exposure), and (iii) drug-related factors (e.g., payload type, linker stability, drug-to-antibody ratio). A summary of these risk factors and their clinical implications is provided in Table 5. This classification may facilitate a more structured approach to risk assessment in clinical practice. Importantly, ADC-related toxicity should not be considered solely as a drug-driven phenomenon, but rather the result of a dynamic interaction between pharmacologic properties and patient-specific susceptibility. This conceptual framework may have relevant implications for the development of predictive models and personalized toxicity risk assessment.

## 6. Risk-Adapted Prevention Strategies

The identification of patients at increased risk of ADC-related toxicities has important clinical implications for prevention and early intervention. In patients receiving trastuzumab deruxtecan, careful baseline assessment of pulmonary function and thorough evaluation of prior lung disease may help identify individuals at higher risk of interstitial lung disease (ILD). In these patients, closer monitoring, including early imaging in the presence of respiratory symptoms, and a lower threshold for treatment interruption may be warranted [53].

Similarly, in patients treated with sacituzumab govitecan, baseline hematologic parameters and prior exposure to myelosuppressive therapies should be considered when evaluating the risk of neutropenia. Prophylactic strategies, including the early use of granulocyte colony-stimulating factors (G-CSF), may be appropriate in selected high-risk patients.

More broadly, integrating clinical risk factors into treatment decision-making may allow for a more personalized approach to ADC therapy, including dose adjustments, intensified monitoring, and early supportive care interventions. Although prospective evidence is limited, a risk-adapted strategy may improve treatment tolerability without compromising efficacy. However, current prevention strategies remain largely empirical and are not yet supported by high-level prospective evidence. The development of validated risk models integrating clinical, biological, and pharmacologic variables represents a key unmet need in this field [73].

## 7. Clinical Management of ADC Toxicities

Effective management of antibody–drug conjugate (ADC)-related toxicities requires a structured, proactive, and multidisciplinary approach, integrating baseline risk assessment, early detection, and timely intervention. Given the heterogeneity of toxicity profiles across ADCs, management strategies should be individualized according to the specific agent, patient-related risk factors, and the type and severity of adverse events [74]. A practical, toxicity-specific management framework is summarized in Table 6.

A cornerstone of clinical management is anticipation and early recognition. Patients should be systematically educated to promptly report early symptoms, such as dyspnea or dry cough (suggestive of interstitial lung disease), diarrhea or persistent nausea (gastrointestinal toxicity), and visual disturbances (ocular toxicity). This patient-centered approach is critical to reduce diagnostic delay and improve outcomes [75].

Baseline assessment plays a key role in risk stratification. Depending on the ADC used, this may include pulmonary evaluation (particularly for trastuzumab deruxtecan), baseline hematologic parameters (especially for sacituzumab govitecan), and cardiovascular assessment in patients with pre-existing risk factors or prior cardiotoxic therapies. This initial evaluation allows for tailored monitoring and facilitates early preventive strategies.

Across all toxicity types, dose interruption, delay, and reduction represent fundamental tools for toxicity management and should be applied according to severity grading and drug-specific prescribing information [76].

### 7.1. Interstitial Lung Disease (ILD)

ILD remains one of the most clinically relevant and potentially life-threatening ADC-related toxicities, particularly with trastuzumab deruxtecan. Management relies on early detection and a grade-based approach.

In grade 1 (asymptomatic) ILD, treatment should be immediately interrupted, and close monitoring should be initiated, including high-resolution CT imaging. Rechallenge may be considered only after complete radiological resolution and careful risk–benefit evaluation.

For grade ≥ 2 ILD, permanent discontinuation of the ADC is required, along with prompt initiation of systemic corticosteroids. Early steroid therapy is critical to prevent progression to severe or fatal disease. Multidisciplinary involvement, including pulmonology consultation, is strongly recommended [77].

### 7.2. Hematologic Toxicity

Hematologic toxicity, particularly neutropenia, is common and often follows recognizable risk patterns, requiring proactive monitoring and supportive care. Management is based on routine blood count monitoring and risk-adapted supportive care.

The use of granulocyte colony-stimulating factors (G-CSFs) is central. Primary prophylaxis may be considered in high-risk patients receiving sacituzumab govitecan, while secondary prophylaxis is recommended in patients experiencing grade ≥ 3 neutropenia or febrile neutropenia.

Dose modifications, including treatment delay and reduction, are effective strategies to maintain safety without compromising efficacy [78].

### 7.3. Gastrointestinal Toxicity

Gastrointestinal toxicity requires a prompt and symptom-driven approach, as delayed intervention may significantly impact patient outcomes.

Early initiation of loperamide is essential for diarrhea, particularly with sacituzumab govitecan. Patients should be educated on self-management strategies and when to escalate care.

For nausea and vomiting, prophylactic antiemetic regimens (including 5-HT3 antagonists, dexamethasone, and NK1 inhibitors) should be administered according to international guidelines.

Supportive care should also include hydration, electrolyte correction, and nutritional support. In refractory cases, additional interventions may be considered [79].

### 7.4. Ocular Toxicity

Ocular toxicity is typically mild but requires early recognition. Preventive strategies include patient education and lubricating eye drops.

Management is mainly supportive, with topical therapies in selected cases. Treatment interruption is rarely required and usually temporary [56].

### 7.5. Stomatitis

Stomatitis should be managed proactively, particularly with newer ADCs such as datopotamab deruxtecan.

Preventive strategies include oral hygiene and avoidance of mucosal irritants, while symptomatic management may involve topical corticosteroids, analgesics, and mouth rinses. Dose modification may be necessary in persistent or severe cases [80].

### 7.6. Cardiovascular Toxicity

Cardiovascular toxicity is less common but increasingly recognized. Management should include baseline cardiovascular assessment and periodic monitoring in high-risk patients.

In cases of suspected cardiotoxicity, treatment interruption and cardiology consultation are recommended. Standard cardioprotective therapies may be initiated as clinically indicated.

Emerging real-world data suggest the need for increased vigilance, particularly regarding thromboembolic events [81].

### 7.7. Hepatotoxicity

Management of ADC-related hepatotoxicity relies primarily on routine laboratory monitoring and the early identification of liver function abnormalities. Baseline assessment should include serum transaminases, bilirubin, and alkaline phosphatase levels, followed by periodic monitoring throughout treatment. In patients experiencing persistent elevations of liver enzymes or bilirubin, treatment interruption and dose modification should be considered according to drug-specific prescribing information. Particular caution is warranted in patients with pre-existing liver disease or concomitant hepatotoxic medications. Rare but serious complications, including nodular regenerative hyperplasia and non-cirrhotic portal hypertension, require specialist evaluation and may necessitate permanent treatment discontinuation [82].

### 7.8. Peripheral Neuropathy

Early recognition of neurologic symptoms is essential to minimize the impact of ADC-related peripheral neuropathy on quality of life and treatment adherence. Patients should be routinely assessed for sensory disturbances, paresthesia, numbness, and motor symptoms, particularly when receiving ADCs carrying microtubule inhibitor payloads. Management is primarily based on treatment interruption and dose reduction according to toxicity severity. Although supportive interventions may provide symptomatic relief, prevention of irreversible neurologic damage remains the primary objective. Early intervention is particularly important because recovery may be prolonged and occasionally incomplete [83].

### 7.9. Infusion-Related Reactions

Most infusion-related reactions are mild to moderate and occur during the first administration. Management includes temporary interruption of the infusion, symptomatic treatment, and close clinical monitoring. Following symptom resolution, treatment can often be resumed at a slower infusion rate. Premedication may be considered in selected patients with previous reactions. Severe hypersensitivity reactions require immediate intervention and may necessitate permanent treatment discontinuation.

### 7.10. Fatigue

Fatigue management should focus on the identification and correction of potentially reversible contributing factors, including anemia, nutritional deficiencies, sleep disturbances, and comorbid conditions. Patient education, physical activity when feasible, psychosocial support, and multidisciplinary interventions may help reduce symptom burden. Because fatigue may significantly affect quality of life despite its generally low grade, systematic assessment should be incorporated into routine clinical practice [84].

### 7.11. Dermatologic Toxicities

Management of dermatologic adverse events is largely preventive and supportive. Patients should receive education regarding skin care measures, including regular use of emollients, avoidance of irritants, and sun protection when appropriate. Mild manifestations may be managed with topical therapies, whereas persistent or severe toxicities may require dermatologic consultation and consideration of dose modification. Early intervention may improve symptom control, treatment adherence, and overall quality of life [85].

### 7.12. Multidisciplinary Approach

Optimal management of ADC-related toxicities requires a multidisciplinary framework involving oncologists and organ-specific specialists. This approach is particularly relevant for complex toxicities such as ILD and cardiovascular events [86].

### 7.13. Closing Statement

Overall, the clinical management of ADC-related toxicities relies on a proactive, personalized, and multidisciplinary strategy. The integration of real-world evidence and the development of standardized management algorithms will be essential to further optimize treatment safety and maximize clinical benefit. Despite the availability of general management principles, the lack of ADC-specific, evidence-based guidelines represents a relevant limitation. Current clinical practice is largely based on extrapolation from conventional chemotherapy and HER2-targeted therapies, highlighting the need for dedicated consensus recommendations and standardized management algorithms.

## 8. Future Perspectives

Future research should focus on the identification and validation of predictive biomarkers of ADC-related toxicity, including circulating markers (e.g., KL-6, surfactant proteins), pharmacogenomic profiles, and imaging-based features [87].

The integration of clinical, biological, and radiological data into predictive models, potentially supported by artificial intelligence, may improve risk stratification and enable more personalized treatment approaches [88].

In parallel, the development of next-generation ADCs with optimized linker stability, reduced systemic exposure, and more selective payloads may further improve the therapeutic index and reduce toxicity [89].

## 9. Conclusions

Antibody–drug conjugates have transformed the treatment landscape of breast cancer and are increasingly used across multiple disease settings. However, their clinical benefit is accompanied by a heterogeneous spectrum of toxicities that extends beyond conventional chemotherapy-related adverse events and may substantially affect treatment delivery, quality of life, and clinical outcomes. Current evidence indicates that ADC-related toxicities arise from a complex interaction between ADC structural characteristics, including target biology, payload properties, linker chemistry, drug-to-antibody ratio, and conjugation strategy, and host-related susceptibility factors. Consequently, toxicity profiles differ substantially across individual ADCs and should not be considered uniform class effects. A mechanistic understanding of these determinants is essential to interpret toxicity heterogeneity, improve monitoring strategies, and support individualized clinical management.

Although important advances have improved the recognition and management of ADC-related adverse events, several challenges remain. Predictive biomarkers are still limited, risk stratification models are incompletely validated, and substantial interindividual variability persists. Future research should focus on integrating clinical, biological, pharmacogenomic, and imaging-based data to improve toxicity prediction and personalize treatment strategies. As ADCs continue to move into earlier treatment settings and broader patient populations, toxicity management will become an increasingly important component of therapeutic decision-making. We propose an integrated framework linking ADC design, toxicity mechanisms, host susceptibility, and clinical management, with the ultimate goal of improving treatment safety while preserving therapeutic efficacy.

## Figures and Tables

**Table 1:** Agent-specific toxicity patterns of ADCs used in breast cancer.

ADC	Target	Payload	Dominant Toxicity Pattern	Key Mechanistic Determinants	Clinical Implications
T-DXd	HER2	DXd	ILD, nausea	High DAR, membrane-permeable payload, bystander effect	Pulmonary monitoring
Sacituzumab govitecan	TROP2	SN-38	Neutropenia, diarrhea	Systemic SN-38 exposure, UGT1A1 metabolism	G-CSF and GI management
T-DM1	HER2	DM1	Thrombocytopenia, hepatotoxicity	Microtubule inhibitor payload, hepatic exposure	Liver monitoring
Datopotamab deruxtecan	TROP2	DXd	Stomatitis	Mucosal epithelial susceptibility	Oral preventive measures

Comparison of currently approved and emerging ADCs in breast cancer according to target antigen, payload class, dominant toxicity profile, underlying mechanistic determinants, and main clinical implications. The table highlights how differences in ADC structure and pharmacologic properties contribute to distinct toxicity patterns, supporting a more individualized approach to toxicity monitoring and management.

**Table 2:** Structural determinants of ADC toxicity and clinical implications.

ADC	Target	Payload	DAR	Linker Type	Key Toxicity	Mechanistic Implication	Clinical Translation
T-DXd	HER2	Topoisomerase I inhibitor	High (~8)	Cleavable	ILD	High DAR + membrane permeability → bystander effect and lung exposure	Requires early detection and prompt steroid treatment
Sacituzumab govitecan	Trop-2	SN-38 (Topo I)	Moderate	Cleavable	Neutropenia, diarrhea	Systemic exposure + UGT1A1 metabolism	Supports proactive G-CSF and early GI management
T-DM1	HER2	DM1 (microtubule inhibitor)	Low (~3.5)	Non-cleavable	Thrombocytopenia	Limited bystander effect	Generally predictable hematologic toxicity
Datopotamab deruxtecan	Trop-2	Topoisomerase I inhibitor	High	Cleavable	Stomatitis	Mucosal epithelial damage	Requires proactive oral care strategies

Overview of key ADC components (target, payload, linker, drug-to-antibody ratio) and their relationship with toxicity profiles and clinical management strategies.

**Table 3:** Mechanistic classification of ADC-related toxicities.

Toxicity Category	Predominant Mechanism	Representative Toxicities	Representative ADCs
Target-mediated toxicity	On-target interaction with antigen-expressing normal tissues	Cardiac dysfunction, epithelial toxicities	HER2- and TROP2-directed ADCs
Payload-mediated toxicity	Off-target cytotoxic effects of released payload and bystander activity	Neutropenia, diarrhea, peripheral neuropathy, stomatitis	T-DXd, SG, T-DM1, Dato-DXd
Linker-mediated toxicity	Premature payload release resulting in systemic exposure	Hematologic and gastrointestinal toxicities	Cleavable-linker ADCs
Platform-mediated toxicity	Fc receptor-mediated uptake, intracellular processing, and catabolite distribution	Hepatotoxicity, thrombocytopenia, pulmonary toxicity *	Multiple ADCs
Host-related susceptibility factors †	Patient-specific biological and clinical determinants modulating toxicity risk	Increased risk of ILD, severe neutropenia, hepatic toxicity	All ADCs

* Pulmonary toxicity, particularly ILD, is likely multifactorial and may involve contributions from payload-, platform-, and host-related mechanisms. † Host-related factors are not direct toxicity mechanisms but may significantly influence toxicity occurrence and severity. Proposed framework for the classification of ADC-related adverse events according to their predominant biological mechanism. Toxicities may arise from target-mediated effects, payload-related cytotoxicity, linker instability, or platform-specific factors, although multiple mechanisms frequently coexist. This classification may facilitate the interpretation of heterogeneous toxicity profiles across different ADCs and support the development of mechanism-based monitoring and management strategies.

**Table 4:** Real-world evidence on ADC-related toxicities in breast cancer.

ADC	Study (Author, Year)	Study Type	N Patients	Setting	Main Toxicity	Key Findings	Clinical Message
T-DXd	Udovica et al., 2026 [35]	Retrospective pooled	1255	Real-world	ILD	Similar incidence vs. trials; higher grade ≥ 3 (2.9% vs. 1.4%)	Severe ILD may be more frequent in clinical practice
T-DXd	Tsurutani et al., post-marketing [34]	Registry	~1700	Post-marketing (Japan)	ILD	~16% ILD; grade ≥ 3 ~3%; grade 5 ~1%	Identifies clinically relevant risk factors
T-DXd	Hourani et al., 2025 [36]	Retrospective	100	Real-world (selected patients)	ILD	Very low incidence of severe ILD	Patient selection influences risk
T-DXd	Botticelli et al., 2024 [37]	Multicenter retrospective study	143	Real-world	ILD	ILD occurred in 2% of patients (3/143); no treatment-related deaths were reported	Safety profile was consistent with clinical trials, with no new safety signals identified
Sacituzumab govitecan	Tam et al., 2026 [25]	Retrospective	87	Real-world	Neutropenia	~49% grade ≥ 3 neutropenia without prophylaxis	High clinical burden; supports G-CSF use
T-DXd and SG	Fountzilas et al., 2025 [38]	Multicenter	312	Real-world	Overall safety	AE rates and toxicity-related discontinuation were consistent with pivotal trials; no new safety signals identified	Supports the reproducibility of ADC safety profiles in routine clinical practice
T-DM1/T-DXd	Aziz et al., 2026 [39]	Real-world database study	31,656	Database	Cardiovascular	Cardiotoxicity occurred in 11.6% (T-DM1) and 9.9% (T-DXd); treatment resumption was associated with improved OS	Supports cardio-oncology management and treatment continuation when feasible

Summary of observational and post-marketing studies evaluating the incidence, severity, and clinical impact of ADC-related toxicities in routine clinical practice.

**Table 5:** Risk factors for ADC-related toxicities and clinical implications.

Category	Risk Factor	Associated Toxicity	Clinical Implication
Patient-related	Older age	ILD, hematologic toxicity	Closer monitoring and dose adaptation
Patient-related	Pre-existing lung disease	ILD	Baseline pulmonary assessment and vigilance
Patient-related	UGT1A1 polymorphism	Neutropenia, diarrhea	Consider genotyping in selected patients
Treatment-related	Prior chemotherapy	Neutropenia	Consider early G-CSF use
Treatment-related	Thoracic radiotherapy	ILD	Increased pulmonary monitoring
Treatment-related	Cumulative exposure	Multiple toxicities	Dose–toxicity relationship; monitor over time

Classification of patient-, treatment-, and drug-related risk factors associated with ADC toxicities, with corresponding implications for clinical risk stratification.

**Table 6:** Practical clinical management of ADC-related toxicities.

Toxicity	Main ADC(s)	Key Risk Factors	Early Signs	First-Line Management	Escalation Strategy	Rechallenge	Key Clinical Warning
Interstitial lung disease (ILD)	T-DXd	Age, prior lung disease, prior RT, Asian ethnicity	Dry cough, dyspnea	Immediate treatment interruption + imaging	Steroids (≥G2), permanent discontinuation	Not recommended if ≥G2	Do not delay evaluation; early steroids improve outcomes
Neutropenia	Sacituzumab govitecan	Prior chemo, baseline cytopenia, UGT1A1	Fever, low ANC	CBC monitoring, G-CSF	Dose reduction, G-CSF intensification	Yes	High incidence requires proactive management
Diarrhea	Sacituzumab govitecan	UGT1A1, GI comorbidities	Diarrhea, dehydration	Early loperamide, hydration	Treatment interruption if ≥G3	Yes	Early treatment prevents severe complications
Nausea/Vomiting	T-DXd	Female sex, prior CINV	Nausea, vomiting	Prophylactic antiemetics	Intensify antiemetics	Yes	Requires upfront prophylaxis
Ocular toxicity	Multiple ADCs	Pre-existing ocular disease	Dry eye, blurred vision	Artificial tears	Topical treatment if needed	Yes	Usually mild but underreported
Stomatitis	Datopotamab deruxtecan	Mucosal fragility	Oral ulcers, pain	Oral hygiene, topical steroids	Dose modification if severe	Yes	Early prevention improves adherence
Cardiovascular toxicity	HER2-ADCs	CV comorbidities, prior anthracyclines	Often asymptomatic	Monitoring, cardiology consult	Treatment interruption	Case-by-case	May be underrecognized in trials

Summary of key toxicities, risk factors, early signs, and recommended management strategies, including escalation approaches and considerations for treatment rechallenge.

## Data Availability

No new data were created in this study. All data discussed in this review are available in the cited publications.

## Abbreviations

The following abbreviations are used in this manuscript:

ADC	Antibody–drug conjugate
ILD	Interstitial lung disease
DAR	Drug-to-antibody ratio
G-CSF	Granulocyte colony-stimulating factor
T-DXd	Trastuzumab deruxtecan
T-DM1	Trastuzumab emtansine
